# Postoperative sepsis-associated neurocognitive disorder: mechanisms, predictive strategies, and treatment approaches

**DOI:** 10.3389/fmed.2025.1513833

**Published:** 2025-06-03

**Authors:** Zijing Gao, Zhenyu Xu

**Affiliations:** ^1^Department of Anesthesiology, The Second Xiangya Hospital of Central South University, Changsha, China; ^2^Department of Infectious Diseases, The Second Xiangya Hospital of Central South University, Changsha, China

**Keywords:** sepsis, sepsis associated encephalopathy, postoperative cognition dysfunction, delium, sepsis treatment

## Abstract

Sepsis is a critical condition characterized by an abnormal immune response to infection, resulting in systemic inflammation, organ failure, and high mortality rate. Postoperative sepsis, accounting for nearly one-third of all sepsis cases, predominantly affects the elderly and individuals with pre-existing conditions, with fatality rates between 30 and 50%. Surgical stress induces immune, hormonal, and metabolic disturbances, heightening susceptibility to immune dysregulation and sepsis. Neurocognitive disorders related to postoperative sepsis, which share pathophysiological similarities with sepsis-associated encephalopathy, involve neuroinflammation, blood–brain barrier disruption, and mitochondrial dysfunction. Cognitive impairments, such as delirium, are frequent postoperative complications that vary in severity depending on the surgical complexity. This review examined the underlying mechanisms of these dysfunctions, the influence of different surgical procedures, and predictive and therapeutic strategies, including machine learning models, aimed at improving patient outcomes.

## 1 Introduction

Sepsis arises from an uncontrolled immune response to infection, resulting in systemic inflammation, organ dysfunction, and elevated mortality rates (1). It remains a significant global health concern, with an estimated 48.9 million cases and 11 million deaths attributed to sepsis annually (2). Despite medical advancements, the economic and healthcare burden continues to escalate, with substantial costs per patient in regions such as the United States, Europe, and China (3–5). Postoperative sepsis, a specific subset occurring after surgical interventions, represents approximately one-third of all sepsis cases and is a major contributor to morbidity and mortality in hospitalized patients. Elderly individuals and those with pre-existing conditions are particularly vulnerable, with mortality rates between 30 and 50%, especially in intensive care settings.

Surgical stress triggers a series of metabolic, hormonal, and immune responses, primarily involving the sympathetic-adrenal-medullary (SAM) and hypothalamic–pituitary–adrenal (HPA) axes (6, 7). These neuroendocrine and immune disturbances exacerbate immune suppression and increase the risk of infection, further aggravating the inflammatory response and fluid imbalances that increase the likelihood of postoperative sepsis (8). This condition significantly affects the brain, leading to complications, such as sepsis-associated encephalopathy (SAE) and postoperative neurocognitive disorders, which are increasingly recognized as severe postoperative outcomes (9–11). Neurocognitive disorders share common pathophysiological features, including neuroinflammation, blood–brain barrier disruption, mitochondrial dysfunction, and cerebral perfusion injury, all of which negatively affect cognitive function (12).

The incidence and severity of postoperative sepsis-associated neurocognitive disorders vary depending on the complexity of the surgery, with more invasive procedures posing higher risks (13). Delirium and cognitive impairments are common manifestations that adversely affect both short-term and long-term recovery (14). These challenges emphasize the need for early detection, prevention, and management strategies. Integrating cognitive assessments, imaging technologies, and advanced predictive tools, such as eXtreme Gradient Boosting (XGBoost) models, could facilitate the identification of high-risk patients and inform therapeutic decision-making (15).

This review examines the underlying mechanisms of postoperative sepsis-associated neurocognitive disorders, evaluates the impact of various surgical procedures, and explores prediction and treatment strategies aimed at improving patient outcomes.

## 2 Sepsis and postoperative sepsis

Sepsis, a critical condition resulting from a dysregulated immune response to infection, remains a global health crisis, contributing to nearly 20% of annual deaths worldwide (16). SAE and related cognitive impairments continue to present significant diagnostic and therapeutic challenges. In cases where sepsis manifests subsequent to a surgical intervention or within the postoperative duration of hospital admission, it is commonly referred to as postoperative or surgical sepsis (17), demarcated as Sepsis develops in individuals undergoing elective surgical interventions with a requisite minimum postoperative hospitalization period of four days (18, 19). It represents approximately one-third of all sepsis cases and is a leading cause of morbidity, multiple organ dysfunction, and mortality in hospitalized patients. It affects 1–3% of surgical patients globally, with a higher prevalence among the elderly and those with pre-existing conditions. Mortality rates are alarmingly high, ranging from 30 to 50%, particularly among ICU patients. Contributing factors include surgical complexity, procedure duration, underlying conditions such as diabetes or immunosuppression, and quality of postoperative care (3, 20, 21).

Surgical stress initiates a complex cascade of metabolic, hormonal, and immune changes, with SAM and HPA axes playing critical roles in maintaining homeostasis (22). Specifically, stimulation of the hypothalamic paraventricular nucleus (PVN) and preoptic area activates the periaqueductal gray matter (PAG), which governs autonomic functions, including cardiovascular and respiratory stress responses (23). Concurrently, activation of the basolateral amygdala (BLA) enhances sympathetic nervous system activity, amplifying cardiovascular responses. These neuroendocrine changes prompt the release of catecholamines and cortisol, which, although intended to restore balance, often exacerbate immune dysregulation (24).

This immune dysregulation can lead to excessive inflammatory response and fluid imbalances, significantly increasing the risk of postoperative sepsis (25). Surgical trauma triggers a biphasic immune response, beginning with an initial pro-inflammatory phase, followed by an anti-inflammatory phase (26). Neutrophils and monocytes rapidly release pro-inflammatory cytokines such as Interleukin-1β (IL-1β), Interleukin-6 (IL-6), Interleukin-8 (IL-8), and Tumor Necrosis Factor-*α* (TNF-α), which fuel inflammatory processes (27). Elevated C-reactive protein (CRP) levels within the first 3–4 days often indicate complications. Natural killer (NK) cell activity diminishes, compromising the body’s ability to defend against infections and tumors. The adaptive immune system is also impaired, as Th1 suppression shifts the Th1/Th2 balance toward Th2 dominance, thereby elevating infection risk (22). This imbalance is further intensified by sustained activation of the SAM and HPA axes, perpetuating the cycle of systemic inflammation and immune suppression.

In contrast to surgical trauma, infections provoke a strong immune response, activating various brain nuclei involved in pathological behavior and immune regulation. Key regions such as the medial preoptic area (MPOA), ventral tegmental area (VTA), nucleus accumbens (NAc), and locus coeruleus (LC) respond to inflammatory cytokines (28, 29). These nuclei then release neurotransmitters, such as serotonin and dopamine, which significantly affect mood, energy levels, and immune function. The hypothalamus plays a vital role in maintaining homeostasis, regulating fever, and orchestrating illness-related behaviors (30).

The activation patterns and neurochemical responses differ markedly between surgical trauma and infection. In surgical trauma, the primary brain regions engaged include the PAG, hypothalamus, and central amygdala (CeA). The neurotransmitters released—glutamate, substance P, and norepinephrine—can suppress immune function and potentially contribute to chronic inflammation (31). This process may amplify local inflammatory responses through nociceptive signaling and sympathetic nervous input to the brain, affecting the PVN and altering neuroendocrine output. Conversely, infection activates the MPOA, VTA, LC, and CeA, releasing neurotransmitters such as serotonin, dopamine, and acetylcholine into the system (28, 32, 33). These agents modulate systemic immune responses through endocrine or autonomic pathways, thereby promoting widespread immune activation. The inflammatory response triggered by infection also heavily engages the HPA axis *via* cytokines, further amplifying the body’s stress response.

Upon the onset of infection within the body or exposure to heightened surgical strain, immune activation occurs, wherein the immune system identifies pathogen-associated molecular patterns (PAMPs) stemming from infections or damage-associated molecular patterns (DAMPs) arising from compromised host cells. The aforementioned molecular patterns engage pattern recognition receptors (PRRs) situated on immune cells, initiating a series of events including the discharge of cytokines and instigation of inflammation, involving signaling components such as Toll-like receptors (TLRs) and nucleotide-binding and oligomerization domain (NOD)-like receptors. Post binding to the recognition domain, the ligand catalyzes, via the effectors domain, signaling pathways that yield outcomes akin to the mobilization and liberation of cytokines, chemokines, hormones, and growth factors, culminating in the onset of a cytokine storm and the activation of the immune system (34–36). PAMPs such as lipopolysaccharide (LPS) and peptidoglycan provoke a robust inflammatory response (37, 38), In contrast, damage-associated molecular patterns (DAMPs) encompass a plethora of molecules, including the renowned high-mobility group box 1 (HMGB1), extracellular cold-inducible RNA-binding protein (eCIRP), adenosine triphosphate (ATP), and enzymatic entities such as nicotinamide adenine dinucleotide (NAD), heat shock proteins (HSPs), histones, members of the S100 family, cell-free DNA (cfDNA), and mitochondrial DNA (mtDNA). These molecules, recognized by numerous immune receptors, potentiate inflammation subsequent to cellular damage caused by surgical interventions or hypoxic conditions (39–41). This immune activation, particularly pronounced in postoperative sepsis, leads to an exaggerated inflammatory state, elevating the risk of organ dysfunction and mortality.

**Figure 1:**
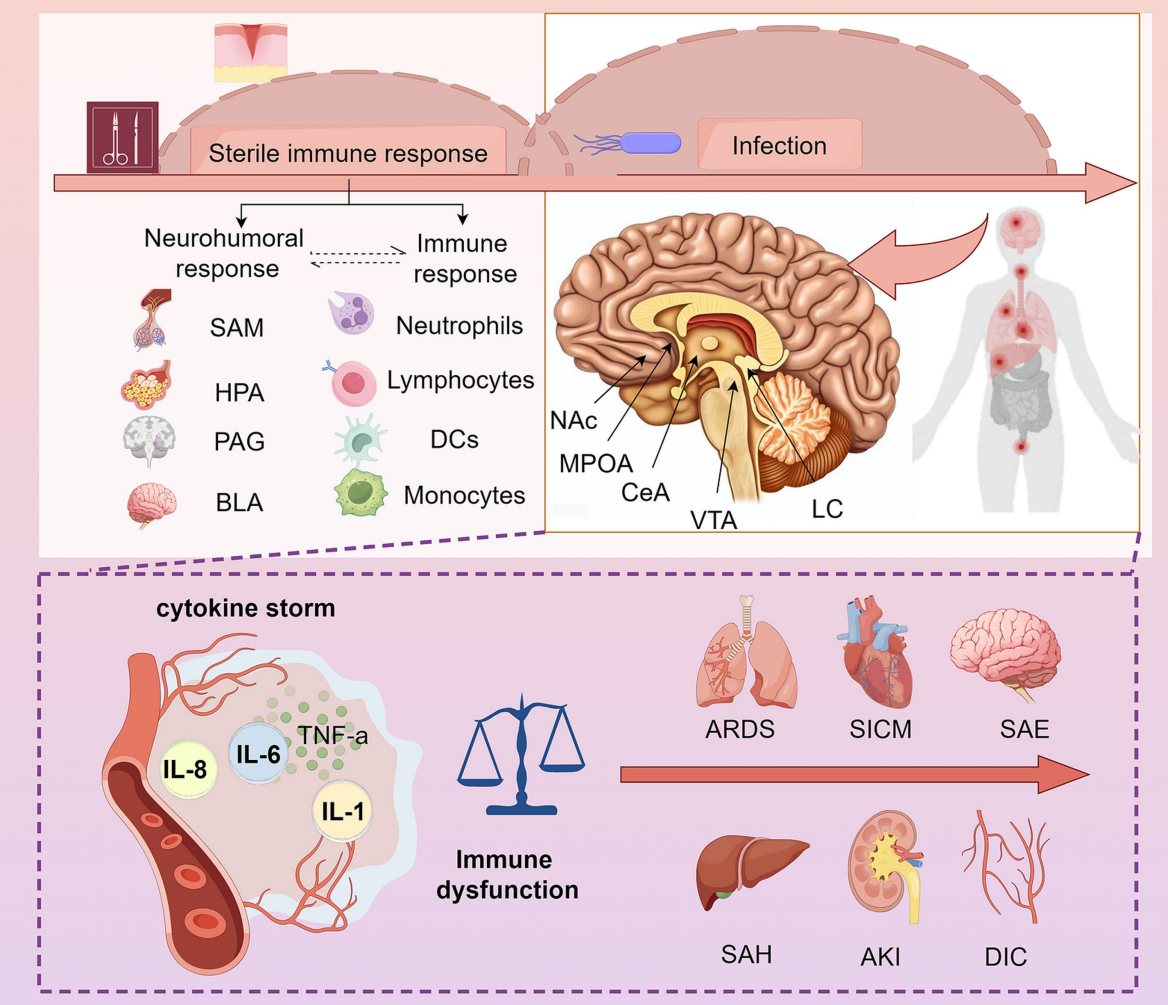
Distinctions and correlations between sepsis and postoperative sepsis. Postoperative sepsis arises specifically from surgical stress, which triggers both neurohumoral and immune responses, with potential interactions between the two. Surgical stress activates neuroendocrine pathways, including the SAM and HPA axes, as well as brain regions such as the PAG and BLA. During the early sterile immune response phase, neutrophils, lymphocytes, NK cells, and monocytes mount a rapid defense. Beyond this phase, both postoperative and non-surgical sepsis involve immune dysregulation driven by infection. An exacerbated immune-inflammatory response can precipitate sepsis, which may subsequently be associated with complications such as ARDS, SICM, SAE, SAH, AKI, or DIC, ultimately leading to multi-organ failure. SAM, Sympathetic Adrenal Medullary; HPA, Hypothalamic Pituitary Adrenal; PAG, Periaqueductal Gray; BLA, Basolateral Amygdala; NAc, Nucleus Accumbens; MPOA, Medial Preoptic Area; CeA, Central Amygdala; VTA, Ventral Tegmental Area; LC, Locus Coeruleus; ARDS, Acute Respiratory Distress Syndrome; SICM, Sepsis Induced Cardiomyopathy; SAE, Sepsis Associated Encephalopathy; SAH, Sepsis Associated Hepatitis; AKI, Acute Kidney Injury; DIC, Disseminated Intravascular Coagulation; IL, Interleukin; TNF-*α*: Tumor Necrosis Factor-alpha. By Figdraw (https://www.figdraw.com/#/).

A diagram (Figure 1) was created to illustrate the correlation between sepsis and postoperative sepsis. Sepsis can arise at any time from various infections, while postoperative sepsis is specifically triggered by surgical interventions and complications such as wound infections. The release of DAMPs from damaged tissues during surgery amplifies the inflammatory response, making postoperative sepsis more severe. Preventing postoperative sepsis requires tailored strategies such as strict adherence to aseptic surgical techniques, diligent wound care, and early infection monitoring.

## 3 The causes of postoperative sepsis- associated neurocognitive disorder

Postoperative sepsis-associated neurocognitive disorders emerge specifically after surgical procedures and are often linked to complications, such as surgical site infections or contamination from instruments. This condition typically presents within the first seven days post-surgery as postoperative delirium (POD), followed by a delayed neurocognitive recovery, characterized by cognitive dysfunction within 30 days, and progressing into postoperative neurocognitive disorder, which can entail cognitive impairment lasting between 30 days and 12 months post-procedure (42, 43). The risk factors are closely associated with the type, method, and duration of the surgery.

Postoperative sepsis-associated neurocognitive disorders share both the pathophysiological mechanisms and clinical manifestations of SAE. Both conditions are neurological complications of sepsis, presenting with similar symptoms such as acute changes in mental status, confusion, disorientation, drowsiness, and, in severe cases, coma or seizures (11, 39, 44). The underlying mechanisms are largely driven by systemic inflammatory responses and metabolic disturbances induced by sepsis. Proposed mechanisms include blood–brain barrier (BBB) disruption, neuroinflammation, and mitochondrial dysfunction (45).

### 3.1 Blood–brain barrier (BBB) changes

The BBB is a selectively permeable and dynamic interface that separates the brain parenchyma from the cerebral circulation and plays a significant role in postoperative sepsis-associated cognitive dysfunction. It is composed of microvascular endothelial cells (ECs), tight junction (TJ) proteins, astrocyte endfeet, pericytes, and capillary basement membrane (46). Within the context of sepsis, systemic elevation of inflammatory cytokines such as IL-1β and TNF-*α* occurs. These cytokines penetrate the central nervous system and act on the BBB to disrupt brain function, leading to perturbations in brain homeostasis and alterations in BBB permeability (47). At the endothelium of the blood–brain barrier (BBB), modifications induced by TNF-*α* lead to the depolymerization of actin, fostering the creation of intercellular gaps within the endothelial cytoskeleton (48). In addition, inflammatory cytokines entering brain tissue can activate microglia, leading to an active microglial phenotype, increased phagocytosis of astrocyte endfeet, and increased BBB permeability. Microglia can decrease paracellular connexin expression, thereby increasing BBB permeability (49).

### 3.2 Mitochondrial dysfunction and oxidative stress

As the most metabolically vibrant organ within the human body, the brain shows substantial oxygen utilization and consumption. Aerobic glucose oxidation stands as the primary fuel source for cerebral activity, with mitochondria assuming a crucial function in orchestrating the oxidative degradation of energy-dense compounds to unleash vitality. In addition to their energy-generating functions, mitochondria actively participate in cellular activities, including calcium balance, production of reactive oxygen species (ROS), and initiation of programmed cell death (50). Increasingly gathered data indicates the pivotal involvement of mitochondria in the onset and progression of sepsis (51). Upon infiltration of the central nervous system by sepsis, the endothelial mitochondrial performance is disrupted. Manifesting as mitochondrial malfunction marked by the interplay of reactive nitrogen species (RNS) and reactive oxygen species (ROS), these dynamic entities inflict harm on cellular constituents, encompassing lipids, proteins, and nucleic acids. Consequently, they impede both mitochondrial respiration and structural integrity (52). Damage to the inner mitochondrial membrane, which results in decreased ATP production, triggers neuronal hypoxic edema, functional deficits, and cognitive impairment. A study focusing on dynamin-related protein 1 (Drp1), a critical protein involved in mitochondrial fission and dysfunction, revealed that the Drp1 inhibitor, P110, mitigated mitochondrial fragmentation and reactive oxygen species (ROS) production, thereby enhancing mitochondrial membrane potential and integrity. Similarly, experimental data indicate that the inhibition of mitochondrial respiration triggered by septic serum can be alleviated using a nitric oxide synthase inhibitor (53, 54).

### 3.3 Neuroinflammation and microglial activation

Microglia, the principal innate immune cells and the first responders within the brain parenchyma, are pivotal in the progression of neurodegenerative diseases (55). They undergo phenotypic alterations across various microenvironments, adopting M1 pro-inflammatory, M2 anti-inflammatory, and other phenotypes (56). M1 microglia are known to provoke neuroinflammation and neuronal apoptosis, whereas M2 microglia are typically stimulated by anti-inflammatory cytokines, such as IL-13 and IL-3, and they secrete IL-10 and neurotrophic factors to aid in the repair of brain tissue and neurons. Cellular receptors, including TLRs and NOD-like receptors are expressed on microglia, enabling them to identify Pathogen-associated molecular patterns (PAMPs) and Damage-associated molecular patterns (DAMPs) (57). In patients with sepsis, microglia are activated by bacteria and other pathogens via TLRs (TLR-2, TLR-4, and TLR-9) and nucleotide-binding oligomerization domain 2 (NOD2). Microglia secrete pro-inflammatory cytokines such as tumor necrosis factor (TNF)-*α*, interleukin (IL)-1β, IL-16, and chemokines such as C-C motif chemokine ligand 2 (CCL2) and IL-18 to attract additional cells and eliminate pathological agents (58). Furthermore, inflammatory cytokines, including IL-1β and IL-6, can activate microglia by crossing the compromised blood–brain barrier (BBB), this persistent neuroinflammation can ultimately lead to neuronal damage or apoptosis (49).

## 4 Postoperative sepsis-associated neurocognitive disorder across different surgeries

Different types and approaches to surgery can provoke and sustain delirium during the postoperative period. For instance, Tavabie et al. (59) studied liver transplant patients and found that those who developed sepsis had a significantly higher risk of neurocognitive disorders, underscoring the need for effective sepsis management in postoperative care. Similarly, Trenschel et al. (60) identified an elevated risk of delirium associated with improper percutaneous endoscopic gastrostomy (PEG) tube placement. Zukowska et al. (61) highlighted the correlation between postoperative neurocognitive disorders and higher infection rates, particularly pneumonia and sternal wound infections. Patients who experienced delirium exhibited markedly reduced 5- and 10-year survival rates, emphasizing the importance of rigorous infection management to improve the long-term outcomes. Moreover, the choice of surgical technique also impacts the incidence of postoperative sepsis and subsequent neurological dysfunction (61). For example, non-cardiopulmonary bypass coronary artery bypass grafting (CABG) has been associated with fewer complications, including lower rates of delirium and sepsis, offering additional benefits to patients with compromised cardiac function (62).

## 5 Prediction of postoperative sepsis-associated neurocognitive disorder

Postoperative sepsis is increasingly recognized as a major factor in the development of Neurocognitive Disorder, particularly among elderly or high-risk surgical patients. Early detection and prediction of sepsis-associated neurocognitive disorder are essential for timely intervention and better clinical outcomes. Building on recent advancements in research on SAE and postoperative cognitive function, integrating cognitive assessment scales, imaging techniques, and biological markers, alongside machine learning algorithms like XGBoost, could significantly enhance the early diagnosis of cognitive impairment associated with postoperative sepsis (Table 1).

**Table 1:** Summary of measures to guide therapy in patients with postoperative sepsis-associated neurocognitive disorder.

Therapeutic interventions	Intervention	Key mechanisms
Infection control		
Empirical antibiotics	Timely administration of broad-spectrum antibiotics	Reduces systemic inflammation and neuroinflammation by controlling infection.
Source control	Surgical debridement/drainage	Eliminate pathogens and reduce tissue infection.
Hemodynamic support		
Fluid resuscitation	Early goal-directed fluid therapy	Restores cerebral perfusion, reduces hypoxia-induced neuronal damage.
Vasopressors	Norepinephrine (target MAP ≥65 mmHg)	Maintains cerebral blood flow, prevents BBB disruption.
Organ support		
Renal replacement therapy (RRT)	Cytokine/endotoxin removal	Reduces circulating inflammatory mediators (e.g., HMGB1, IL-6) driving neuroinflammation.
Immunomodulation		
Corticosteroids	Hydrocortisone (200 mg/day)	Suppresses excessive neuroinflammation, stabilizes BBB.
Adjunctive therapies		
Haloperidol	Low-dose haloperidol for agitation	Modulates dopamine signaling; limited efficacy in delirium resolution.
Dexmedetomidine	Sedation with dexmedetomidine	Inhibits TLR-4/NF-κB pathway; enhances hippocampal neuroprotection via PI3K/Akt signaling.
Emerging therapies		
Recombinant BNP	rhBNP administration	Improves cardiac output and cerebral perfusion; reduces oxidative stress.
Oxytocin	Oxytocin infusion	Suppresses microglial activation via OXTR/ERK/STAT3 pathway; preserves synaptic function.
Ferroptosis inhibitors	Irisin or NAC	Attenuates oxidative stress and mitochondrial dysfunction via SIRT1/Nrf2 pathways.
Resveratrol	Resveratrol supplementation	Antioxidant and anti-inflammatory effects enhances mitochondrial biogenesis.

### 5.1 Cognitive evaluation

Mental status changes are commonly screened using a variety of assessment tools. For sedated patients, scales such as the Glasgow Coma Scale (GCS), Full Outline of Unresponsiveness (FOUR) score, and Richmond Agitation-Sedation Scale (RASS) are widely used, with a modified GCS available for pediatric populations (63–65). In non-sedated patients, the Adaptation to the Intensive Care Environment (ATICE) scale is effective for assessing consciousness and comprehension based on visual stimuli responses (66). Additionally, delirium is often evaluated using the Confusion Assessment Method (CAM) and its ICU-specific counterpart (CAM-ICU), which assess four key dimensions: consciousness, attention, disorganized thinking, and clarity of awareness (67).

Concerning frailty and postoperative outcomes, Mahanna et al. (68) observed that frail or prefrail patients, identified *via* the FRAIL scale, were more prone to POD, even when adjusted for baseline cognitive function. However, frailty was not linked to an increased risk of postoperative cognitive decline (POCD) in older adults undergoing noncardiac surgeries. This finding suggests that while frailty may increase vulnerability to delirium, it does not necessarily contribute to long-term cognitive decline following noncardiac procedures.

### 5.2 Radiomics

#### 5.2.1 Electroencephalography (EEG) and transcranial Doppler ultrasonography (TCD)

EEG and TCD ultrasound are critical tools for understanding and managing POD, a condition characterized by acute brain dysfunction and poor surgical outcomes (69, 70). In POD, EEG commonly shows a shift toward lower frequencies, indicating delirium-related encephalopathy. Serial or continuous EEG monitoring enhances the detection of delirium and can uncover epileptic causes (71). Despite its recognized potential, further research is needed to establish reliable EEG markers and underlying mechanisms of POD. Notably, Fritz et al. (72) found that EEG suppression at lower anesthetic levels during surgery was linked to a higher POD risk (35%) compared to typical suppression (17%). Additionally, TCD ultrasound provides a non-invasive method to estimate cerebral blood flow by monitoring velocity, though its effect on patient outcomes remains uncertain, with up to 10% of patients having inadequate acoustic windows for TCD (73).

#### 5.2.2 Magnetic resonance imaging (MRI)

Postoperative delirium is closely related to structural brain changes and specific imaging characteristics. Studies suggest that septic delirium can cause hyperintensities in the hippocampus on diffusion-weighted imaging (DWI) MRI, resembling changes seen in global hypoxia (74). Additionally, the detection of increased perivascular spaces (PVS) in the centrum semiovale on brain MRI within six months before surgery is strongly associated with a heightened risk of POD, especially in older adults (75). Although regional anesthesia does not significantly reduce the incidence of delirium compared to general anesthesia, preoperative cortical thinning and increased postoperative EEG delta power correlate with greater delirium severity (76). Moreover, an increase in ventricular size is significantly linked to delirium following cardiac surgery, suggesting that cerebral atrophy may elevate susceptibility to POD. Both preoperative and postoperative brain MRI characteristics can thus be valuable in identifying high-risk patients and facilitating early intervention to mitigate POD risk (77).

#### 5.2.3 Computed tomography (CT)

Delirium, a common and severe condition, is often linked to significant cerebral metabolic disturbances, as revealed by imaging studies like FDG PET scans. Haggstrom et al. (78) found that older inpatients with delirium exhibited widespread, reversible cortical hypometabolism, particularly in the posterior cingulate cortex (PCC), which plays a pivotal role in attention—a central feature of delirium. This hypometabolism was correlated with inattention and the duration of delirium, suggesting its role in the cognitive impairment observed during and after delirium episodes.

However, the connection between structural brain changes and POD remains unclear. Cavallari et al. (79) reported that preoperative brain atrophy and white matter hyperintensities (WMHs) were not significantly associated with the incidence or severity of POD in older patients without dementia. Their study found no significant differences in MRI-derived measures between patients who developed delirium and those who did not, indicating that preexisting cerebral structural changes may not predispose patients to POD or exacerbate its severity in non-demented individuals. While metabolic changes in the brain during delirium are evident and linked to cognitive impairment, structural changes like brain atrophy and WMHs observed through CT scans may not reliably predict POD in older adults without dementia.

### 5.3 Biomarkers

#### 5.3.1 Cytokines

Perioperative neurocognitive disorders (PND), including postoperative delirium, are closely associated with neuroinflammation, where cytokines play a pivotal role. Smith et al. (80) identified elevated levels of CXCL1, CXCL10, IL-8, IL-1 receptor antagonist, and IL-10 in ICU patients with delirium, with cytokine profiles varying according to triggers such as sepsis, COVID-19, or surgery. IL-1, IL-6, TNF-*α*, and IL-15 are particularly implicated in postoperative delirium. Kimura et al. (81) demonstrated that elevated postoperative IL-15 levels are linked to organ dysfunction and poor outcomes in patients with sepsis, while Pavcnik Arnol et al. (82) showed significant post-surgical increases in IL-6, underlining its role in neuroinflammation monitoring.

#### 5.3.2 Inflammatory markers

CRP has been widely studied as a biomarker for predicting postoperative cognitive dysfunction (POCD), including delirium, particularly in older adults (83, 84). Persistently elevated CRP levels beyond postoperative day 4 are strongly associated with higher delirium risk, especially in APOE ε4 allele carriers, suggesting a genetic predisposition to inflammation-driven cognitive impairment (84). CRP kinetics can thus serve as a valuable tool in identifying high-risk patients, enabling closer monitoring and tailored management (85).

Procalcitonin (PCT) has emerged as a critical biomarker for forecasting postoperative sepsis and related complications. Booka et al. (86) revealed that elevated PCT levels following esophagectomy correlate with a higher risk of infections and poor long-term prognosis. Elevated PCT has also been linked to postoperative sepsis-associated neurocognitive disorder, highlighting its dual significance in assessing both infection and cognitive impact.

HMGB1 plays a critical role in neuroinflammation and postoperative delirium. Studies by Terrando et al. (87) established that elevated HMGB1 levels are associated with postoperative memory deficits and neuroinflammation, which can be mitigated by blocking HMGB1 activity (40). Yin et al. (39) further confirmed HMGB1’s role in SAE, where it contributes to synaptic loss and cognitive impairment, positioning it as a potential therapeutic target for preventing postoperative neurocognitive disorders.

#### 5.3.3 Immune cells

Recent advancements underscore the importance of immune cell subsets in predicting postoperative sepsis-associated neurocognitive disorders. Lymphocyte depletion, particularly of CD4 and CD8 subsets, correlates with poorer outcomes, including recognition dysfunction. Monitoring PD-1^+^ NK cells has shown promise as a prognostic biomarker, with evidence linking them to increased 28-day mortality (88). Additionally, the neutrophil-to-lymphocyte ratio (NLR) has gained recognition as a key marker, with higher NLR values associated with worse outcomes in SAE.

#### 5.3.4 NeuroMarkers

Neuron-specific enolase (NSE) and neurofilament light chains (NfL) are well-established markers of neuronal injury (89, 90). EEG patterns indicative of moderate to severe encephalopathy correspond with elevated serum levels of NSE and NfL. NSE, a dimeric isoenzyme of enolase, has been linked to delirium, with decreased cerebrospinal fluid (CSF) NSE and increased CSF lactate levels suggesting a metabolic shift from aerobic to anaerobic processes (78). Elevated serum NfL levels are similarly associated with disease activity, progression, and response to therapy in Alzheimer’s disease (AD).

### 5.4 Machine learning

Ren et al. (91) developed and validated the MySurgeryRisk artificial intelligence system, which predicts postoperative complications using electronic health record (EHR) data. Yao et al. (15) demonstrated that the XGBoost model outperformed stepwise logistic regression in predicting in-hospital mortality for postoperative patients with sepsis, identifying key predictors such as fluid-electrolyte disturbances, coagulopathy, and renal replacement therapy. This system exhibited consistent performance in predicting various complications, including organ injury, neurological complications, and mortality, with high accuracy in both retrospective and prospective settings. Similarly, Marra et al. (92) developed a dynamic risk model capable of predicting daily fluctuations in ICU patients’ acute brain dysfunction, such as delirium and coma, showing high predictive accuracy for outcomes including delirium and mortality. This model enhances ICU care by facilitating the anticipation of patient needs and guiding interventions.

**Figure 2:**
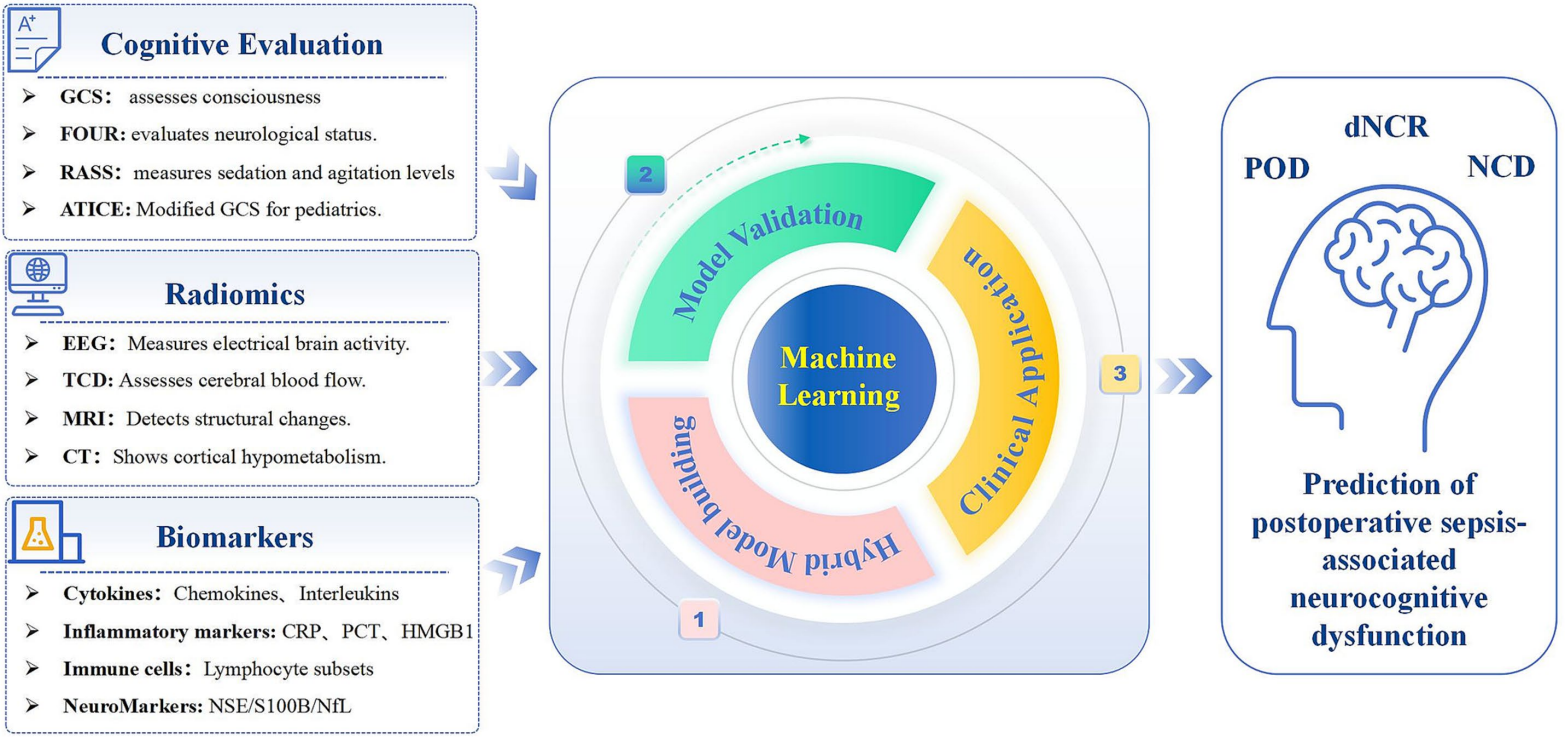
Conceptual framework for a hybrid model predicting postoperative sepsis-associated neurocognitive disorder. This hybrid model integrates clinical assessments, biomarkers, imaging studies, and machine learning techniques to predict postoperative sepsis-associated neurocognitive disorder. By incorporating cognitive evaluation tools (e.g., CAM-ICU), radiomics (e.g., EEG, MRI), and biomarkers (e.g., cytokines), the model provides real-time risk assessments. Machine learning algorithms, such as XGBoost, analyze these inputs, facilitating early diagnosis and customized interventions for patients at risk. GCS, Glasgow Coma Scale; FOUR, Full Outline of Unresponsiveness; RASS, Richmond Agitation-Sedation Scale; ATICE, Adaptation to the Intensive Care Environment; EEG, Electroencephalography; TCD, Transcranial Doppler; MRI, Magnetic Resonance Imaging; CT, Computed Tomography; NSE, Neuron-Specific Enolase; S100B, S100 calcium-binding protein B; NfL, Neurofilament light chain.

By integrating data from these models, a hybrid machine-learning approach can be developed to predict postoperative sepsis-associated neurocognitive disorder. This prediction tool would combine biochemical markers, imaging data, and clinical grading systems, delivering real-time risk assessments akin to Ren et al.’s MySurgeryRisk system and Marra et al.’s dynamic risk model. Such a tool could enable early identification of at-risk patients and guide targeted treatments, ultimately improving postoperative outcomes (Figure 2).

## 6 Treatment of postoperative sepsis-associated neurocognitive disorder

Postoperative sepsis-associated neurocognitive disorder remains a relatively underexplored area, with most research focused on SAE. Since both conditions share similar mechanisms in later stages, advancements in SAE treatment could inform therapeutic strategies for postoperative sepsis-associated neurocognitive disorder. This section will review the latest developments in sepsis management, including fluid resuscitation and antimicrobial therapy, and their potential applications in treating neurocognitive dysfunction associated with postoperative sepsis.

### 6.1 Clinical treatment

#### 6.1.1 Fluid resuscitation

Postoperative patients frequently require fluid therapy to address surgical stress and volume loss, and in the context of postoperative sepsis, early fluid resuscitation becomes even more critical. Rapid initial fluid administration can restore overall circulation, including cerebral perfusion, potentially preventing or alleviating sepsis-associated neurocognitive disorder (93). Early improvements in cerebral circulation are vital for mitigating the cognitive decline commonly associated with postoperative sepsis. Resuscitation strategies should extend beyond normalizing blood pressure, focusing on broader physiological targets such as improving capillary refill time, lactate clearance, and urinary output (94).

#### 6.1.2 Antimicrobial therapy

Timely and appropriate antimicrobial therapy is another cornerstone of sepsis management, including postoperative sepsis. Studies demonstrate that early initiation of antibiotics significantly improves outcomes, as delays are closely linked to increased mortality (95). Prompt antimicrobial treatment not only controls systemic infection but also plays a critical role in preventing or limiting the progression of SAE and postoperative neurocognitive disorder (44). By swiftly managing the infection, antibiotics help reduce systemic inflammation and neuroinflammation, both of which are key contributors to cognitive impairment in patients with sepsis.

#### 6.1.3 Vasoactive drugs

Vasopressor support is essential for maintaining adequate perfusion pressure in septic shock. The recommended mean arterial pressure (MAP) target is generally 65 mmHg, though individualized adjustments may be necessary for patients with pre-existing hypertension (96). Norepinephrine is the preferred vasopressor due to its effectiveness and lower risk of inducing arrhythmias. Ensuring sufficient cerebral perfusion is crucial in postoperative sepsis, as poor blood flow exacerbates neuroinflammation and disrupts the blood–brain barrier, further contributing to neurocognitive dysfunction (97). Combining early vasopressor use with fluid resuscitation and antimicrobial therapy may help reduce neurological complications in affected patients.

#### 6.1.4 Extracorporeal blood purification

Extracorporeal blood purification is an emerging approach to managing the dysregulated immune response in sepsis, including SAE. By filtering out a broad range of inflammatory mediators, this technique helps lower cytokine levels below harmful thresholds, thereby reducing local tissue damage and mitigating systemic inflammation. In addition to removing endotoxins and PAMPs, it restores immune balance (98). Reducing cytokine storms and enhancing immune function can protect the brain from neuroinflammatory damage, offering potential benefits in managing SAE and postoperative sepsis-associated neurocognitive disorder (99).

#### 6.1.5 Corticosteroids

Corticosteroids have been studied as adjunctive therapy in sepsis (100), particularly in the context of SAE, where they may mitigate neuroinflammation by suppressing excessive immune responses, lowering cytokine levels, and stabilizing the blood–brain barrier (101). This modulation of the inflammatory cascade could reduce neurological damage and improve outcomes for patients with SAE and postoperative sepsis-associated neurocognitive disorder.

#### 6.1.6 Dexmedetomidine

Dexmedetomidine has gained recognition as a promising agent in the management of PNDs and delirium in critically ill patients (102). Previous studies have shown that systemic administration of dexmedetomidine enhances neurocognitive functions. It achieves this by inhibiting the TLR-4/NF-κB pathway while activating the hippocampal neuronal PI3K/Akt/GSK3β pathway, promoting neuroprotection (87). This anti-inflammatory mechanism is also linked to a reduction in sepsis-induced cognitive decline, making dexmedetomidine a valuable therapeutic option for addressing neuroinflammation-related cognitive impairment (103).

#### 6.1.7 Haloperidol

The use of haloperidol in managing delirium in critically ill patients remains a topic of debate. Smit et al. (104) reported that haloperidol, whether administered alone or with clonidine, was associated with a lower likelihood of delirium resolution and longer delirium duration, with no impact on ICU mortality. This suggests haloperidol may not shorten delirium duration and could even prolong it. Conversely, Duprey et al. (105) found that treating incident delirium with haloperidol resulted in a dose-dependent reduction in 28-day and 90-day mortality, indicating potential survival benefits despite ongoing debate about its efficacy in resolving delirium. While haloperidol may offer survival advantages for delirious ICU patients, its effectiveness in treating delirium and its impact on overall ICU outcomes warrant further investigation.

### 6.2 Animal experiments

#### 6.2.1 Recombinant human brain natriuretic peptide (rhBNP)

rhBNP has emerged as a promising treatment for sepsis-induced cardiac dysfunction, improving cardiac function by lowering NtproBNP and cTnI levels while enhancing left ventricular ejection fraction (LVEF). Beyond its cardiovascular benefits, rhBNP’s capacity to improve systemic circulation could positively influence cerebral perfusion, potentially benefiting patients with SAE (106). By stabilizing overall hemodynamics, rhBNP may contribute to mitigating neurological damage in SAE.

#### 6.2.2 Oxytocin

Oxytocin has shown potential in addressing cognitive and memory dysfunction in SAE by modulating neuroinflammation. It exerts neuroprotective effects by inhibiting microglial activation *via* the OXTR/ERK/STAT3 pathway, thereby preserving hippocampal synaptic function (107). This anti-inflammatory action positions oxytocin as a potential therapeutic agent for improving neurological outcomes in sepsis.

#### 6.2.3 Ferroptosis inhibitors/irisin

Ferroptosis inhibitors, particularly irisin, have demonstrated efficacy in reducing organ damage and may also target SAE. By activating the SIRT1/Nrf2 pathway and suppressing ferroptosis-related proteins such as GPX4, irisin reduces inflammation and oxidative stress, potentially preventing neuronal damage in SAE (108).

#### 6.2.4 Resveratrol

Resveratrol, known for its anti-inflammatory and antioxidant properties, offers neuroprotective benefits in sepsis through the activation of the SIRT1/Nrf2 pathway. By reducing oxidative stress and improving mitochondrial function, resveratrol may help preserve cognitive function in patients with sepsis (109). Its protective actions on organs and its anti-inflammatory and effects suggest that it could play a vital role in preventing neurocognitive decline associated with sepsis.

#### 6.2.5 Erythropoietin (EPO)

EPO, traditionally used to manage sepsis-associated anemia, may also benefit SAE by improving oxygen delivery and reducing ischemic brain injury. By stimulating erythropoiesis and increasing hemoglobin levels, EPO enhances cerebral oxygenation, potentially mitigating cognitive impairment in SAE (110).

#### 6.2.6 N-acetylcysteine (NAC)

Free radical generation and oxidative stress are critical factors in sepsis-induced brain damage (111). Studies on sepsis models have shown that NAC, either alone or in combination with other agents, can inhibit reactive oxygen species (ROS) production, elevate brain antioxidant levels, and attenuate neuroinflammation (112). Additionally, a combination of NAC and an iron chelator has been found to reduce neuronal loss and restore Na^+^, K^+^-ATPase activity, essential for maintaining resting potential, ion transport, neuronal cell volume, cognitive function, and overall brain signal transduction.

#### 6.2.7 Sevoflurane

The anesthetic sevoflurane has been crucial in reducing sepsis-induced apoptosis. In a sepsis model, sevoflurane mitigates cognitive dysfunction by suppressing the NLRP3-dependent caspase-1/11-GSDMD pathway-mediated pyroptosis in the hippocampus through the upregulation of SIRT1. Furthermore, by modulating the caspase 3/9 and Bax/Bcl signaling pathways, potential interventions may target sepsis-associated encephalopathy and memory impairment. Rg1, a significant component of ginseng, has also been shown to protect the hippocampus from SAE (113).

## 7 Conclusions and perspectives

Postoperative sepsis-associated neurocognitive disorder arises from the complex interplay between systemic inflammation, immune dysregulation, and neural injury, all exacerbated by surgical trauma (22, 26). The release of DAMPs and heightened neurohormonal responses in the surgical context add additional layers to the pathophysiology, making postoperative patients—particularly those with preexisting conditions or undergoing high-risk surgeries—more vulnerable to cognitive decline (37). While these mechanisms share similarities with SAE, the surgical setting introduces distinct challenges that necessitate targeted management strategies.

POCD is a frequent complication of anesthesia, particularly among older adults. Factors such as age, educational background, preoperative cognitive status, and comorbidities influence its occurrence. A large-scale prospective study found that each surgical procedure can be linked to a slight cognitive decline, with reductions in hippocampal volume and increased white matter hyperintensities (114). Interestingly, bariatric surgery has shown long-term cognitive improvements in obese individuals, likely due to weight loss, hormonal shifts, decreased systemic inflammation, and gut microbiota alterations.

Postoperative pain is another factor contributing to cognitive decline, as it can impact brain regions involved in learning and memory (75, 115). Animal studies have demonstrated that postoperative pain can decrease the expression of N-methyl-D-aspartate receptor subunits in the hippocampus, resulting in cognitive impairment (116). Moreover, postoperative pain often exacerbates preexisting sleep disturbances, further impairing cognitive function (32). Effective perioperative pain management, including regional anesthesia techniques, may help reduce these risks and improve early postoperative cognitive outcomes (117).

Patients undergoing surgery are often in a state of immune priming, which increases their susceptibility to postoperative infections. When these infections progress to sepsis, the combined effects of immune dysregulation and systemic inflammation can lead to more severe neurocognitive deficits (118). Although the clinical presentations of postoperative delirium and SAE may overlap, postoperative sepsis carries a higher risk of poor outcomes due to the added burden of comorbidities and inflammatory responses. This underscores the need for heightened vigilance and tailored management strategies in these patients.

In conclusion, while surgery poses significant risks to cognitive function, understanding the intricate relationship between surgical trauma and infectious processes is essential. Targeted interventions, deeper insights into the underlying mechanisms, and ongoing research into the effects of different surgical procedures on neural integrity are essential for improving cognitive outcomes post-surgery. This review aims to provide clinicians with a comprehensive understanding of postoperative sepsis-associated neurocognitive disorder, including key predictive factors and treatment strategies, to enhance care and outcomes for this vulnerable population.
